# Precision medicine in action for Pompe disease

**DOI:** 10.1016/j.omtn.2024.102265

**Published:** 2024-07-09

**Authors:** Antonietta Tarallo, Giancarlo Parenti, Nicola Brunetti-Pierri

**Affiliations:** 1 Department of Translational Medicine, Section of Pediatrics, University of Naples Federico II, Naples, Italy; 2 Telethon Institute of Genetics and Medicine, Pozzuoli, Italy; 3 Scuola Superiore Meridionale (SSM, School of Advanced Studies), Genomics and Experimental Medicine Program, University of Naples Federico II, Naples, Italy

## Main text

Glycogen storage disease type II, more often referred to as Pompe disease, is an inherited metabolic disorder caused by deficiency of lysosomal acid α-glucosidase (GAA). The disease primarily affects skeletal and cardiac muscles. Patients are classified as having infantile-onset Pompe disease (IOPD) or late-onset Pompe disease (LOPD) based on the age at which clinical manifestations appear. More than 650 pathogenic variants in the gene encoding GAA have been reported. While the intronic variant c.-32-13T>G is found in approximately 80%–90% of patients with LOPD, most patients carry private variants. Enzyme replacement therapy (ERT) for Pompe disease has markedly improved patient survival, but central nervous system involvement refractory to ERT has been observed in aging patients.^1^ Despite initial improvement after the initiation of ERT, some patients show clinical decline, and muscle involvement is resistant to treatment. Furthermore, the recombinant enzyme used for ERT is immunogenic, and patients who are cross-reactive immune material negative are especially at risk of developing anti-GAA antibodies that result in decreased ERT efficacy or severe immune reactions. The limitations of ERT have driven the development of several preclinical studies and ongoing gene therapy clinical trials for LOPD and IOPD.

In preclinical models, gene therapy has been performed *in vivo* using adeno-associated virus (AAV) vectors targeting either the affected muscles or the liver as a depot for the production and secretion of GAA.^2^ Muscle-directed delivery of GAA has resulted in long-term expression in muscle cells but requires higher vector dosages. In contrast, the doses needed for liver-directed AAV-based gene therapy are lower, but the efficacy of treatment is shorter if vector administration is performed early in life because of the loss of episomal AAV vector genomes due to cell division. Therefore, strategies based on vector genome integration or genome editing are more attractive for sustained disease correction. Genome editing by CRISPR-Cas9 requires double-strand DNA breaks (DSBs) that lead to undesired consequences, including uncontrolled gene insertions or deletions and chromosomal abnormalities.

Base editors are DSB-independent methods that can result in C>T conversions (cytidine base editors) or A>G conversions (adenine base editors [ABEs]). Christensen and colleagues used ABEs in human skin fibroblasts from three patients with IOPD for the correction of three pathogenic nonsense variants in the gene encoding GAA.^3^ The degree of correction of the target variants was up to 96%, which resulted in detectable GAA protein and enzyme activity within the normal range. While the efficiency of the adenine correction in this proof-of-concept *in vitro* study is impressive, significant preclinical work still needs to be performed for the clinical development of base editing for Pompe disease therapy. The delivery of the ABE machinery to a large fraction of muscle cells required for correction of Pompe disease is challenging. Because the limited AAV packaging capacity precludes the use of full-length base editors, dual AAVs delivering split ABEs that are reconstituted in the target cells by *trans*-splicing inteins are required.^4^ However, in preclinical studies, optimized dual AAVs enabled *in vivo* base editing with only 9% efficacy in skeletal muscle.^5^ Although Duchenne muscular dystrophy gene therapy supports the possibility of correcting several muscle cells, there are also risks of life-threatening toxicity related to the high doses of AAV needed to achieve this goal.^6^ Nevertheless, novel muscle-tropic AAV vectors developed by a directed evolution strategy^7^ have the potential to transduce a higher percentage of muscle fibers. In addition to their increased tropism for both skeletal and cardiac muscles, both highly desirable for Pompe disease treatment, these novel AAV vectors show a liver de-targeting effect, a feature that is less attractive for Pompe disease compared to congenital muscle dystrophies.

Although the risks for ABE-mediated mutagenesis are expected to be lower than CRISPR-Cas9, ABEs are only needed transiently to provide the machinery for editing, and long-term expression is not desirable. Expression of ABEs might also induce an immune response against corrected cells. Small-molecule switching elements can be used to overcome the problems of long-term ABE expression. Alternatively, lipid nanoparticle (LNP)-mediated delivery of the gRNA and RNA for ABEs is an attractive option for short-term expression. However, LNPs that specifically and efficiently target skeletal muscles, including the diaphragm, which is an important muscle target in Pompe disease, require further development and optimization.^8^

Patients with IOPD under ERT show neurocognitive impairment during the first years of life and slowly progressive cerebral white matter abnormalities. A muscle-specific therapeutic approach is not expected to address this disease complication. Another major practical challenge is the development of mutation-specific ABEs in a disorder with hundreds of different pathogenic variants. Developing humanized mouse models carrying each of the target mutations does not appear to be a practical solution. Nevertheless, platform technology programs might streamline the approval of products based on reproducible technology with the same vector, delivery method, and mode of action. Despite the shortcomings and the extensive work still needed to generate *in vivo* proof-of-concept efficacy, the work by Christensen and colleagues^3^ is setting the stage for the future development of base editing for Pompe disease that has potential for safe and long-term disease correction.
